# Awareness of and reactions to health and environmental harms of red meat among parents in the United States

**DOI:** 10.1017/S1368980021003098

**Published:** 2022-04

**Authors:** Anna H Grummon, Dina Goodman, Lindsay M Jaacks, Lindsey Smith Taillie, Christina A Chauvenet, Meg G Salvia, Eric B Rimm

**Affiliations:** 1 Harvard Center for Population and Development Studies, Harvard TH Chan School of Public Health, Cambridge, MA, USA; 2 Department of Population Medicine, Harvard Medical School and Harvard Pilgrim Health Care Institute, Boston, MA, USA; 3 Department of Global Health, Harvard TH Chan School of Public Health, Boston, MA, USA; 4 Global Academy of Agriculture and Food Security, The University of Edinburgh, Edinburgh, UK; 5 Department of Nutrition, University of North Carolina Gillings School of Global Public Health, Chapel Hill, NC, USA; 6 Carolina Population Center, University of North Carolina at Chapel Hill, Chapel Hill, NC, USA; 7 Arnold School of Public Health, University of South Carolina, Columbia, SC, USA; 8 Department of Nutrition, Harvard TH Chan School of Public Health, Boston, MA, USA; 9 Department of Epidemiology, Harvard TH Chan School of Public Health, Boston, MA, USA

**Keywords:** Meat, Environmental sustainability, Messages, Communication, Parents, Food labelling

## Abstract

**Objective:**

Evidence of the health and environmental harms of red meat is growing, yet little is known about which harms may be most impactful to include in meat reduction messages. This study examined which harms consumers are most aware of and which most discourage them from wanting to eat red meat.

**Design:**

Within-subjects randomised experiment. Participants responded to questions about their awareness of, and perceived discouragement in response to, eight health and eight environmental harms of red meat presented in random order. Discouragement was assessed on a 1-to-5 Likert-type scale.

**Setting:**

Online survey.

**Participants:**

544 US parents.

**Results:**

A minority of participants reported awareness that red meat contributes to health harms (ranging from 8 % awareness for prostate cancer to 28 % for heart disease) or environmental harms (ranging from 13 % for water shortages and deforestation to 22 % for climate change). Among specific harms, heart disease elicited the most discouragement (mean = 2·82 out of 5), followed by early death (mean = 2·79) and plants and animals going extinct (mean = 2·75), though most harms elicited similar discouragement (range of means, 2·60–2·82). In multivariable analyses, participants who were younger, identified as Black, identified as politically liberal, had higher general perceptions that red meat is bad for health and had higher usual red meat consumption reported being more discouraged from wanting to eat red meat in response to health and environmental harms (all *P* < 0·05).

**Conclusions:**

Messages about a variety of health and environmental harms of red meat could inform consumers and motivate reductions in red meat consumption.

High consumption of meat, particularly red and processed meat, increases risk of CVD, diabetes and some cancers^(1–8)^. Further, red meat is a major contributor to environmental harms such as greenhouse gas emissions^(1,9–13)^, air and water pollution^(1,9)^, biodiversity loss^(1,14)^ and deforestation^(14,15)^. Reducing red meat consumption is therefore an important strategy for reducing chronic disease risk and mitigating environmental damage^(16)^.

Despite growing recognition of the health and environmental harms of red meat, American adults consume an average of 284 g/week (about 0·6 pounds) of unprocessed red meat alone (i.e. not including processed red meats such as bacon), nearly three times the maximum level recommended for optimising human and planetary health^(2)^. More than half of Americans say they are willing to eat less red meat^(17)^. Yet red meat consumption is projected to increase over the next decade^(18)^, perhaps in part because many Americans are unaware of the health and environmental harms of red meat^(17,19)^. Given this willingness to change, coupled with lack of awareness about red meat’s impacts, educating consumers about the harms of red meat could reduce red meat consumption.

A growing body of research has shown that communicating about a products’ health harms, for example, through product warning labels and mass media campaigns, is an effective strategy for reducing unhealthy behaviours including cigarette smoking^(20,21)^, alcohol consumption^(22)^ and sugary drink consumption^(22–25)^. Similarly, a recent systematic review found that providing information about the health effects of meat consumption is an effective strategy for reducing intentions to eat meat as well as meat consumption^(26)^. Emerging literature also suggests the promise of communicating about products’ environmental harms as a strategy for changing consumer behaviour. For example, a randomised experiment with undergraduate students in the UK found that sending students 2 weeks of daily messages about the environmental effects of meat production reduced students’ red and processed meat consumption compared with a no-message control group^(27)^. What remains unknown is which specific health and environmental harms hold the most promise for motivating consumers to reduce their red meat intake. Identifying the specific harms that most discourage red meat intake is important because messaging campaigns may not be able to communicate about all harms (e.g. due to space constraints), and because prior studies of tobacco and sugary drink messages suggest advantages to shorter, simpler messages^(28–32)^. Also unknown is whether consumers’ reactions to health and environmental harms of red meat vary by demographic characteristics, information that could help to tailor messages to specific groups.

To inform communication efforts, we examined consumers’ responses to health and environmental harms of red meat in an experiment with US parents of young children. Parents are a critical group to study in dietary communication interventions because their behaviours influence both their own health and the dietary habits of their children^(33)^. Parents of young children (i.e. under age five) are especially important, given that dietary habits in early childhood affect diet and health later in childhood and into adolescence^(34,35)^. Moreover, US parents are nearly 40 % less likely than non-parents to have reduced their red meat intake compared with 3 years ago^(19)^, suggesting red meat reduction campaigns may be especially beneficial for this group. Thus, the specific objectives of this study were to examine which health and environmental harms of red meat parents are aware of and which are most likely to discourage red meat consumption. Additionally, to provide insight on populations that might respond more strongly to messages about red meat’s harms, we examined demographic predictors of awareness of health and environmental harms and of the extent to which these harms discouraged participants from wanting to consume red meat.

## Methods

Prior to data collection, we pre-registered the sample size, hypotheses and analysis plan (https://aspredicted.org/q5e9b.pdf). The only deviations from this plan were that we corrected for multiple comparisons using Bonferroni’s method rather than Tukey’s method because Tukey’s method cannot be applied to mixed models, and that we conducted two unplanned exploratory analyses, examining: (1) predictors of awareness of health and environmental harms and (2) predictors of harm-induced discouragement separately for health *v*. environmental harms.

### Participants

In January 2020, we recruited a convenience sample of 544 US adults using the survey research firm Dynata as part of a study of parents’ responses to experimental stimuli and survey questions. Participants were eligible if they were aged 18 years or older and had a child aged 6 months to 5 years. Online convenience samples can provide efficient and generalisable findings for experiments like the one used in this study^(36)^.

### Procedures

Participants provided informed consent, completed an online survey programmed in Qualtrics and received incentives from Dynata (e.g. gift cards, reward points).

### Measures

**Fig. 1:**
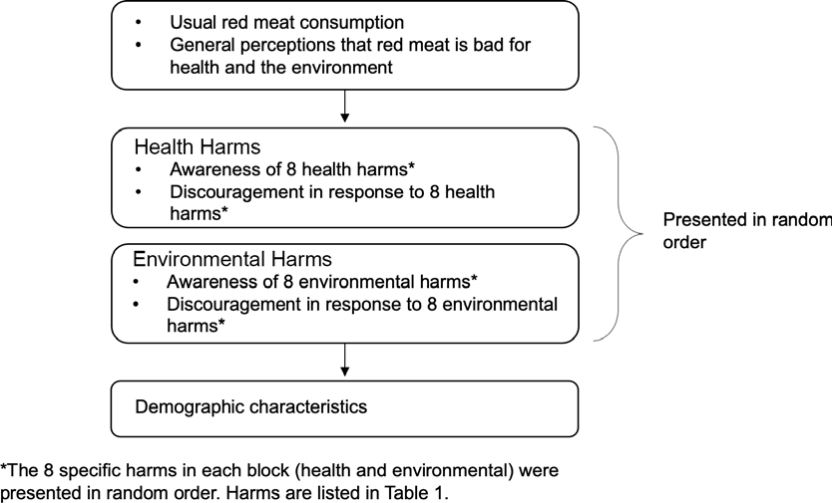
Flow chart of survey questions

A flow of survey questions is shown in Fig. 1. First, participants answered questions about their usual red meat consumption^(19,37)^ and general perceptions that red meat is bad for health and for the environment (e.g. ‘How bad or good for your health do you think eating red meat is?’). Next, they responded to questions about their awareness of, and discouragement in response to, specific health and environmental harms of red meat. The order of presentation of health and environmental harms was randomised such that half of participants answered questions about health harms first and half answered questions about environmental harms first.

We assessed awareness of harms using a select-all-that-apply question adapted from previous studies^(38–40)^, ‘Before today, had you ever heard that eating red meat can contribute to the following harms?’ Then, we listed the eight health or eight environmental harms, displayed in random order. Participants could also select ‘I haven’t heard of red meat contributing to any of these harms before;’ this option was always displayed last.

Next, we assessed the extent to which each harm discouraged participants from wanting to eat red meat using an item adapted from previous studies^(38–40)^, ‘How much does knowing that eating red meat contributes to these harms discourage you from wanting to eat red meat?’ We assessed perceived discouragement because meta-analytic evidence indicates that perceived message effectiveness is predictive of actual message effectiveness^(41)^. Participants rated perceived discouragement in response to each harm on a 5-point scale, from ‘Not at all’ (coded as 1) to ‘Very much’ (coded as 5).

We selected harms to present based on recent literature linking red meat production and consumption with health and environmental harms (Table 1). The eight health harms were type 2 diabetes^(42,43)^, weight gain^(43–45)^, heart disease^(43,46)^, stroke^(47,48)^, colon cancer^(43,49,50)^, prostate cancer^(49)^, stomach cancer^(49)^ and early death^(43,51)^. The eight environmental harms were climate change^(14,15)^, more greenhouse gases^(1,9–12)^, water shortages^(1,11,52)^, water pollution^(1)^, air pollution^(9)^, plants and animals going extinct^(1,14)^, clearing of forests^(14,15)^ and worse land quality^(1,15,53)^. All participants rated their awareness and discouragement for each of the eight health harms and each of the eight environmental harms. Within each type of harm (health *v*. environmental), specific harms were presented in random order.

**Table 1:** Health and environmental harms of red meat shown in experiment and supporting evidence

Red meat harms	Supporting evidence
Health harms	
Type 2 diabetes	• Meta-analyses have found that both unprocessed and processed red meat intake are associated with increased risk of type 2 diabetes^(3,72)^
Weight gain	• A systematic review^(45)^ and a meta-analysis^(44)^ found that red and processed meat intake is associated with weight gain and increased risk of overweight and obesity
Heart disease	• Meta-analyses have found that red meat intake is associated with CVD risk factors^(46)^ and increased risk of CHD and heart failure^(73)^
Stroke	• Meta-analyses have found that red meat intake is associated with increased risk of stroke^(47,73)^
Colon cancer	• Meta-analyses have found that red and processed meat intake is associated with increased risk of colorectal cancer^(4,74,75)^. Additionally, the International Agency for Research on Cancer (IARC) classifies red meat as probably carcinogenic to humans^(5)^
Prostate cancer	• A meta-analysis found that red meat intake is associated with increased risk of prostate cancer^(5)^ • A pooled analysis of fifteen prospective cohorts found that red and processed meat intake is associated with increased risk of advanced prostate cancer^(76)^
Stomach cancer	• A meta-analysis found that red meat intake is associated with increased risk of gastric (i.e. stomach) cancer^(77)^
Early death	• A meta-analysis found that red meat intake is associated with increased risk of all-cause mortality^(78)^ • A pooled analysis of two prospective cohorts found that red meat intake is associated with increased risk of all-cause mortality^(79)^
Environmental harms	
Climate change	• Systematic reviews of life cycle analyses indicate that production of red meat is a major contributor to greenhouse gas emissions^(9,14)^, which are a key driver of climate change^(80)^
More greenhouse gases	• Systematic reviews of life cycle analyses indicate that production of red meat is a major contributor to greenhouse gas emissions^(9,14)^ • Ruminant (e.g. cow, goat, sheep) production is a major contributor to methane emissions^(80,81)^
Water shortages	• Production of red meat is a major contributor to water use^(82)^ and water scarcity (i.e. the relative freshwater availability in a given region)^(83)^
Water pollution	• Production of red meat is a major contributor to water pollution, including through leaching of fertilisers and pesticides used to grow animal feed^(82)^ and through increases in eutrophication^(9)^ (the process by which water becomes enriched with minerals and nutrients, stimulating algal blooms and other negative ecological effects)
Air pollution	• Systematic reviews of life cycle analyses indicate that production of red meat is a major contributor to acidifying emissions (e.g. SO_2_, NH_3_ and NO_*x*_)^(9)^
Plants and animals going extinct	• Meat production (particularly red meat) contributes to biodiversity loss through habitat destruction (e.g. when land is converted to use for feed production or animal grazing, or due to nutrient pollution)^(1,84,85)^
Clearing of forests	• Red meat (particularly beef) production is a major contributor to deforestation (e.g. when forests are converted to pasture for cattle)^(14,86)^
Worse land quality	• Red meat production contributes to land degradation via overgrazing, compaction and erosion^(15,87)^

Finally, the survey assessed standard demographics. Survey measures appear in online supplementary material, Supplemental Exhibit 1.

### Analysis

First, we calculated the proportion of participants who reported they were aware of each harm and the mean discouragement ratings for each harm. We also calculated the proportion of participants who were aware of at least one harm, and the mean number of harms for which participants indicated awareness, both overall and separately for health and environmental harms. Next, we assessed whether likelihood of reporting awareness of harms was higher for health compared with environmental harms using mixed effects logistic regression, regressing awareness (coded as 0/1 for each harm) on an indicator variable for whether the harm was a health or environmental harm. These models treated the intercept as random to account for repeated measures within participants.

To shed light on the populations who were most aware of red meat’s harms, analyses also examined demographic predictors of the total number of harms for which participants indicated awareness (summed across all sixteen health and environment harms). These analyses used negative binomial regression with robust standard errors. We regressed the total number of harms for which participants indicated awareness on the following potential predictors: age, gender, race/ethnicity, educational attainment, income, political leaning and usual red meat consumption. We also used this approach to examine predictors of awareness separately for health harms *v*. environmental harms.

Next, analyses assessed whether health or environmental harms were more effective at discouraging participants from wanting to eat red meat. These analyses used a linear mixed model, regressing harm-induced discouragement ratings on an indicator variable for whether the harm was a health or environmental harm, treating the intercept as random. We then assessed the extent to which each specific harm elicited discouragement using a linear mixed model with indicator variables for each of the sixteen harms (excluding one as the referent), again treating the intercept as random. We used *z*-tests to conduct pairwise comparisons of predicted mean discouragement for each harm, applying Bonferroni’s method to adjust for multiple comparisons. These comparisons allowed us to determine which of the harms (if any) were more discouraging than the others while adjusting for repeated measures within participants.

To examine which population groups reported more discouragement in response to health and environmental harms of red meat, we also examined demographic predictors of average discouragement from wanting to eat red meat. First, we averaged discouragement ratings across all sixteen harms. We then used ordinary least squares linear regression to assess predictors of average discouragement. These analyses assessed the same demographic predictors as for awareness and additionally examined general perceptions that red meat is bad for health and for the environment. Exploratory analyses used the same approach to examine predictors of average harm-induced discouragement separately for health harms *v*. environmental harms.

Analyses were conducted in 2021 using Stata MP version 16.1 (StataCorp LLC).

## Results

Participants’ average age was 33·8 (sd 8·0) years (range: 19–80). About two-thirds were White (69 %), 18 % were Latino(a), 8 % were another race/ethnicity and 5 % were Black (Table 2). Slightly more than half (57 %) of participants identified as female and 22 % had a high school education or less.

**Table 2:** Participant characteristics, *n* 544 US parents of young children

Characteristic	*n*	%
Age in years		
18–25	91	17
26–34	209	39
35–44	204	38
45+	38	7
Gender		
Male	227	42
Female	308	57
Non-binary	1	0·2
Race/ethnicity		
Non-Hispanic White	373	69
Non-Hispanic Black or African American	29	5
Hispanic	97	18
Non-Hispanic other	45	8
Education		
High school or less	120	22
Some college	85	16
College degree	224	41
Graduate degree	114	21
Annual household income		
Less than $25 000	90	17
$25 000–$49 999	106	19
$50 000–$74 999	90	17
$75 000–$99 999	115	21
$100 000 or more	143	26
Political party		
Liberal	136	25
Moderate	218	40
Conservative	189	35
General perceptions of how good or bad red meat is for health		
Very bad	29	5
Somewhat bad	78	14
Neither good nor bad	221	41
Somewhat good	135	25
Very good	80	15
General perceptions of how good or bad red meat is to the environment		
Very bad	29	5
Somewhat bad	82	15
Neither good nor bad	272	50
Somewhat good	88	16
Very good	73	13
Usual red meat intake (servings/d)		
Mean	0·7	
sd	0·8	

Missing data ranged from 0·0% to 1·5%.

For each of the sixteen harms, fewer than one-third of participants indicated awareness that red meat contributed to that harm (Table 3). About one-third (33 %) of participants were not aware of any of the sixteen harms; 46 % were not aware of any of the health harms, and 51 % were not aware of any of the environmental harms. The specific harms with the highest level of awareness in the sample were heart disease (28 % reported awareness), weight gain (27 %), climate change (22 %) and increased greenhouse gas emissions (21 %). Participants were least aware that red meat contributes to stomach cancer (11 %) and prostate cancer (8 %). In mixed effects logistic regression, participants were similarly likely to report awareness of harms regardless of harm topic (health *v*. environment, OR = 1·02, *P* = 0·77).

**Table 3:** Awareness and discouragement of the health and environmental harms of red meat consumption, *n* 544 US parents of young children

	Awareness	Discouragement	
Harm of red meat	%	Mean	sd
Health harms			
Heart disease	28	2·82	1·46
Weight gain	27	2·75	1·45
Stroke	17	2·75	1·45
Colon cancer	14	2·74	1·46
Type 2 diabetes	13	2·68	1·44
Early death	13	2·79	1·49
Stomach cancer	11	2·72	1·44
Prostate cancer	8	2·60	1·47
Environmental harms			
Climate change	22	2·70	1·44
Greenhouse gas emissions	21	2·70	1·44
Water pollution	18	2·69	1·42
Air pollution	16	2·67	1·42
Land quality	16	2·65	1·42
Extinction of plants and animals	14	2·75	1·45
Deforestation	13	2·68	1·43
Water shortages	13	2·68	1·45

In multivariate analyses examining predictors of the number of harms for which participants reported awareness, participants aged 26–34 years reported awareness of about 0·3 fewer harms of red meat compared with those aged 18–25 years (B = –0·33, *P* = 0·029, Table 4). Participants who identified as female reported being aware of fewer harms than those who identified as male (B = –0·26, *P* = 0·023). Likewise, those who identified as politically moderate (B = –0·31, *P* = 0·013) or conservative (B = –0·34, *P* = 0·011) reported awareness of fewer harms than those who identified as liberal. Participants who identified as Black reported awareness of more harms than those identifying as White (B = 0·57, *P* = 0·006), but identifying as Latino(a) or as another race/ethnicity (compared with identifying as White) was not associated with awareness. Higher educational attainment and higher income were generally associated with being aware of more harms. Usual red meat consumption was not associated with being aware of more health and environmental harms of red meat (B = 0·04, *P* = 0·558).

**Table 4:** Associations between participant characteristics and the total number of health and environmental harms of red meat for which participants reported awareness, *n* 544 US parents of young children

	B	se	*P*
Age in years			
18–25	Reference		
26–34	**−0·33**	**0·15**	**0·029**
35–44	−0·16	0·16	0·304
45 or older	−0·03	0·21	0·886
Female*	**−0·26**	**0·11**	**0·023**
Race/ethnicity			
White	Reference		
Black	**0·57**	**0·21**	**0·006**
Latino(a)	0·07	0·14	0·622
Other race/ethnicity	0·11	0·14	0·455
Education			
High school or less	Reference		
Some college	0·29	0·21	0·176
College degree	**0·51**	**0·18**	**0·003**
Graduate degree	**0·52**	**0·21**	**0·013**
Annual household income			
Less than $25 000	Reference		
$25 000–$49 999	0·37	0·20	0·062
$50 000–$74 999	**0·42**	**0·19**	**0·028**
$75 000–$99 999	**0·40**	**0·20**	**0·045**
$100 000 or more	**0·41**	**0·20**	**0·046**
Political leaning			
Liberal	Reference		
Moderate	**−0·31**	**0·13**	**0·013**
Conservative	**−0·34**	**0·13**	**0·011**
Red meat consumption, servings per day	0·04	0·06	0·558

Bs are unstandardised regression coefficients from negative binomial regressions, regressing the total number of health and environmental harms for which participants reported awareness on participant characteristics. Models estimated robust standard errors. Bold coefficients are statistically significant, *P* < 0·05.

* Referent group was male. The one non-binary participant was excluded from analysis due to small cell size.

In analyses of awareness of health harms only, participants who identified as Black (compared with White) and those who had higher educational attainment reported awareness of a greater number of health harms. By contrast, participants who identified as politically moderate (compared with liberal) reported awareness of fewer harms (online supplementary material, Supplemental Table 1). In analyses of awareness of environmental harms only, being age 26–34 years (compared with 18–25), identifying as female (compared with male) and identifying as politically conservative (compared with liberal) were associated with awareness of fewer harms (online supplementary material, Supplemental Table 2). Higher education, higher income and higher usual red meat consumption were associated with awareness of more of the environmental harms of red meat.

**Fig. 2:**
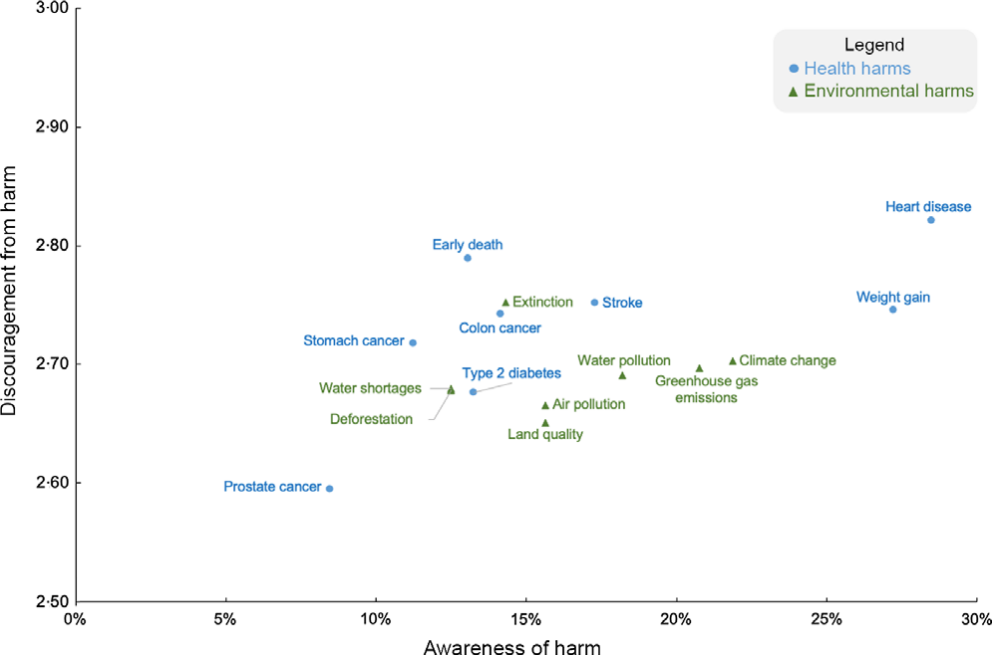
Health and environmental harms of red meat by discouragement and awareness, *n* 544 US parents of young children. 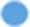, health harms; 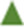, environmental harms

In mixed effects regressions of harm-induced discouragement, health harms elicited slightly more discouragement than environmental harms, but the magnitude of the difference was small (mean discouragement 2·73 *v*. 2·69 on the 1–5 Likert scale; B = 0·04, *P* = 0·010). Harms with higher awareness generally elicited higher discouragement (Fig. 2). Among specific harms, heart disease elicited the highest mean discouragement (mean = 2·82 on the 1–5 Likert scale), followed by early death (mean = 2·79), plants and animals going extinct (mean = 2·75), stroke (mean = 2·75) and weight gain (mean = 2·75) (Table 3). Prostate cancer (mean = 2·60) and worse land quality (mean = 2·65) elicited the lowest discouragement. After adjusting for multiple comparisons, the only significant differences in discouragement between harms were that early death was more discouraging than prostate cancer (difference in predicted means = 0·20, adjusted *P* = 0·002), and heart disease was more discouraging than both prostate cancer (difference = 0·23, adjusted *P* < 0·001) and worsening land quality (difference = 0·17, adjusted *P* = 0·024).

In multivariate analyses examining predictors of average discouragement ratings, older participants generally reported lower discouragement than younger participants (Table 5). Participants who identified as Black reported higher levels of average discouragement compared with White participants (B = 0·56, *P* = 0·019). Those who identified as politically moderate (B = –0·40, *P* = 0·002) or conservative (B = –0·64, *P* < 0·001) were less discouraged by the health and environmental harms of red meat than those who identified as politically liberal. Participants who reported higher general perceptions that red meat is bad for health also reported higher average discouragement (B = 0·18, *P* = 0·006). By contrast, general perceptions that red meat is bad for the environment were not associated with average discouragement (B = 0·07, *P* = 0·329). Finally, participants who reported higher usual red meat consumption reported higher average discouragement (B = 0·36, *P* < 0·001). Gender, education, income and other race/ethnicities (i.e. Latino(a) and other race/ethnicity) were not associated with average discouragement (all *P*s > 0·05). The pattern of results was similar when examining harm-induced discouragement separately for health *v*. environmental harms (online supplementary material, Supplemental Tables 3 and 4).

**Table 5:** Associations between participant characteristics and average discouragement from wanting to eat red meat in response to health and environmental harms of red meat across, *n* 544 US parents of young children

	B	se	*P*
Age in years			
18–25	Reference		
26–34	**−0·40**	**0·16**	**0·011**
35–44	**−0·41**	**0·16**	**0·013**
45 or older	−0·26	0·24	0·272
Female*	−0·04	0·12	0·724
Race/ethnicity			
White	Reference		
Black	**0·56**	**0·24**	**0·019**
Latino(a)	0·22	0·14	0·119
Other race/ethnicity	−0·20	0·19	0·281
Education			
High school or less	Reference		
Some college	0·12	0·17	0·489
College degree	0·29	0·16	0·063
Graduate degree	0·32	0·20	0·109
Annual household income			
Less than $25 000	Reference		
$25 000–$49 999	0·09	0·18	0·607
$50 000–$74 999	0·07	0·19	0·702
$75 000–$99 999	0·32	0·20	0·113
$100 000 or more	0·07	0·20	0·750
Political leaning			
Liberal	Reference		
Moderate	**−0·40**	**0·13**	**0·002**
Conservative	**−0·64**	**0·14**	**< 0·001**
General perceptions that red meat is bad for health	**0·18**	**0·07**	**0·006**
General perceptions that red meat is bad for the environment	0·07	0·07	0·329
Red meat consumption, servings per day	**0·36**	**0·08**	**< 0·001**

Bs are unstandardised regression coefficients from ordinary least squares regressions regressing participants’ average discouragement ratings (across all sixteen health and environmental harms) on participant characteristics. Bold coefficients are statistically significant, *P* < 0·05.

* Referent group was male. The one non-binary participant was excluded from analysis due to small cell size.

## Discussion

Results of this study suggest that messages describing the health and environmental harms of red meat could be a promising strategy for discouraging red meat consumption among US parents of young children. The majority of respondents were not yet aware of the specific health and environmental harms of red meat assessed in this study, and one-third were not aware of *any* of the sixteen harms examined. These results suggest a major opportunity to educate consumers and motivate positive behaviour change. Expectancy disconfirmation theory posits that when consumers receive negative information about a product that conflicts with their prior expectations (e.g. being informed about the harms of red meat when they had previously not known these harms), their attitudes towards the product will become more negative^(54–56)^. This theory would suggest that correcting consumers’ misperceptions about the health and environment risks of red meat could motivate them to reduce their red meat consumption. In line with this prediction, one study found that warning messages about the health harms of sugary drinks led to larger changes in parents’ attitudes and purchase intentions when the messages were displayed on beverages parents had perceived as healthier compared with beverages parents already understood to be unhealthy^(57)^.

Regression analyses revealed that participants who were 26–34 years old (compared with 18–25 years), identified as female (compared with male) and identified as politically moderate or conservative (compared with liberal) reported awareness of fewer harms of red meat. By contrast, participants who identified as Black (compared with White) and those with higher educational attainment and higher income reported being aware of more harms. These results suggest that it may be beneficial to tailor awareness-raising campaigns to particular groups with lower awareness, such as parents who identify as female, are politically moderate or conservative or have lower educational attainment or income. However, given that the majority of participants were unaware that red meat contributes to the health and environmental harms assessed in this study, educational efforts are likely to benefit parents from all demographic groups.

Participants’ usual red meat consumption was not related to their awareness of red meat’s health harms, but higher red meat consumption did predict higher awareness of the environmental harms of red meat. The reason for this association is unclear. One explanation is that higher red meat consumers are more likely to pay attention to information about the environmental consequences of red meat consumption because this information is particularly relevant to them, but have not yet acted on their awareness by reducing their red meat consumption. Regardless of the explanation, this finding highlights that interventions would likely benefit from incorporating a variety of strategies to reduce red meat consumption, including increasing the accessibility, availability and attractiveness of non-meat options^(58)^.

Several participant characteristics predicted higher discouragement from wanting to eat red meat in response to health and environmental harms. For example, consumers aged 18–25 years reported higher discouragement in response to environmental harms of red meat compared with those aged 26–34 and 35–44 years, perhaps because young adults have stronger interest in environmental sustainability and greater concern about climate change than older adults^(59–61)^. Younger adults also reported higher discouragement in response to health harms than older adults. This pattern of results differs somewhat from prior research finding that younger adults were less likely than older adults to report health reasons for not eating meat^(62)^. Our results could potentially reflect a growing openness among young adults towards reducing their red meat consumption or consuming a plant-forward diet^(63)^, regardless of the precise motivation for making dietary changes. Participants who reported higher red meat consumption also reported higher discouragement in response to health and environmental harms of red meat. This finding is encouraging, as it suggests that messages about the harms of red meat might have the greatest impact on those who stand to benefit the most from reducing their red meat intake. Additionally, participants who had stronger general perceptions that red meat is bad for health reported being more discouraged, on average, in response the specific health and environmental harms examined in this study. By contrast, general perceptions that red meat is bad for the environment were not associated with average discouragement ratings. These findings might suggest that strengthening the public’s *general* perception that red meat is bad for health could increase the public’s receptivity to messages about *specific* health or environmental harms. However, the observed associations between participants’ characteristics and their average discouragement ratings should be interpreted with caution because we cannot rule out the possibility that some demographic groups (such as young adults or those who hold stronger general perceptions that red meat is bad for health) might respond more strongly to *any* type of message presented in an online survey, even messages not about red meat. Studies that experimentally compare red meat messages to control messages are needed to establish whether characteristics like age, meat consumption, and general perceptions about red meat influence the effectiveness of red meat reduction messages on consumer behaviour.

Communication interventions such as product warnings and mass media campaigns that describe the health harms of cigarettes^(20,21)^, alcohol^(22)^ and sugary drinks^(22–25)^ have been shown to generate small but meaningful reductions in purchases and consumption of these products, suggesting that communicating about the harms of red meat could help curb red meat intake. We found that both health and environmental harms elicited similar levels of perceived discouragement. Likewise, a variety of health and environmental harms were similarly discouraging to consumers. These results suggest that message developers have many promising options for topics to address in meat reduction messages. The limited differences in mean discouragement observed between the specific harms also suggest that communication campaigns could easily rotate among these harms, a strategy that could help prevent messages from becoming ‘stale’ and losing efficacy over time^(64)^.

The strengths of this study include the comprehensive set of health and environmental harms tested and the experimental comparison of how much each harm motivated participants to reduce their red meat consumption. Limitations include the use of a convenience sample of parents and the relatively young age distribution of the sample. Although prior studies indicate that online convenience samples can provide similar experimental results as probability samples^(36,65,66)^, future research should confirm our findings with non-parents and with a wider range of ages. Additionally, although we did not query whether participants were vegetarian or vegan, about 13 % of our sample reported eating red meat less than 1 time per week during the past 30 d. Future studies may wish to examine awareness and discouragement specifically among non-vegans/vegetarians or among high red meat consumers, for whom messages may be most relevant. Another limitation is that we did not assess whether some consumers believe that red meat is *beneficial* for specific health and environmental outcomes; understanding whether these beliefs are widespread, and who is most likely to hold these beliefs, could help inform messaging campaigns. Additionally, this study focused on health and environmental harms because these are two of the key reasons that US adults report as motivating them to change their diet or reduce their meat consumption^(19,67)^. However, consumers might also be motivated by other harms of red meat production. For example, a recent meta-analysis suggested that interventions appealing to animal welfare (e.g. by portraying farm animals) hold promise for reducing meat purchases and consumption^(68)^. We also did not assess other potentially important aspects of message design, such as message framing^(69–71)^. Finally, while perceived message effectiveness is predictive of behaviour change^(41)^, we did not assess behavioural outcomes. Future studies should experimentally evaluate the extent to which messages describing different types of harms of red meat reduce red meat purchases and consumption.

## Conclusions

Reducing meat consumption is critical for mitigating climate change and reducing chronic disease burden^(16)^. Our study suggests that communication interventions describing how red meat consumption affects both human and planetary health hold promise for informing US consumers and motivating reductions in red meat consumption.

## Supplementary material

For supplementary material accompanying this paper visit https://doi.org/10.1017/S1368980021003098. click here to view supplementary material
