# Supplementary material for: Awareness of and reactions to health and environmental harms of red meat among parents in the United States
Source: Public Health Nutr. 2021 Jul 29;25(4):893–903. doi: 10.1017/S1368980021003098 (PMC8799779; doi:10.1017/S1368980021003098)
Supplement: Supplementary file 1 [file S1368980021003098sup001.docx]

**Awareness of and reactions to health and environmental harms of red meat among parents in the United States**

**Online Supplemental Material**

**Supplemental Exhibit 1.** Survey measures used in study

**Supplemental Table 1.** Associations between participant characteristics and number of health harms of red meat for which participants indicated awareness, *n*=544 US parents of young children

**Supplemental Table 2.** Associations between participant characteristics and number of environmentala harms of red meat for which participants indicated awareness, *n*=544 US parents of young children

**Supplemental Table 3.** Associations between participant characteristics and average discouragement from wanting to eat red meat in response to health harms of red meat, *n*=544 US parents of young children

**Supplemental Table 4.** Associations between participant characteristics and average discouragement from wanting to eat red meat in response to environmental harms of red meat across, *n*=544 US parents of young children

**Supplemental Exhibit 1.** Survey measures used in study

| **Construct** | **Item** | **Response scale** | | **Source** |
| --- | --- | --- | --- | --- |
|  | **Meat Awareness/Discouragement Items** | |  | |
| Prompt | The next questions are about red meat. Red meat refers to beef, lamb, pork, sausage, and ham. Red meat does NOT include chicken, turkey, or seafood. |  | |  |
| Red meat consumption | In the past 30 days, how often did you eat red meat? | 0=Never or less than 1 time per week  1=1 time per week  3=2-4 times per week  5.5=5-6 times per week  7=1 time per day  14=2 times per day  21=3+ times per day | | Adapted from Neff et al. 2018^(1)^ and NHANES Dietary Screener Questionnaire 2009-2010 |
| Perceived health harmfulness of red meat | [randomize order of health and environmental harmfulness]  How bad or good for your health do you think eating red meat is? | 1=Very bad  2=Somewhat bad  3=Neither good nor bad  4=Somewhat good  5=Very good | |  |
| Perceived environmental harmfulness of red meat | How bad or good for the environment do you think eating red meat is? | 1=Very bad  2=Somewhat bad  3=Neither good nor bad  4=Somewhat good  5=Very good | |  |
|  | **Awareness/Discouragement** | |  | |
| Shared prompt | The next items will ask you about possible harms from eating red meat.  [Randomize order of Health & Environment blocks] |  | |  |
|  | **Health** | |  | |
| Harm awareness  (Prompt) | Before today, had you ever heard that eating red meat can contribute to the following harms?  (Check the harms that you had heard about below) | [Check all that apply; randomize order of stimuli within health/environmental sections, “I haven’t heard” is always last.] | | Adapted from Rohde et al. 2019^(2)^ |
| Health harms | 1. Type 2 diabetes 2. Weight gain 3. Colon cancer 4. Heart disease 5. Stroke 6. Prostate cancer 7. Stomach cancer 8. Early death   I haven’t heard of red meat contributing to any of these harms before [make answer exclusive] |  | |  |
| Harm discouragement – Health harms | [page break]  Eating red meat may expose people to the harms we just asked you about.  How much does knowing that eating red meat contributes to these harms discourage you from wanting to eat red meat?  [Show stimuli in a matrix table, randomized order] | [program as matrix table]  1=Not at all  2=A little  3=Somewhat  4=A lot  5=Very much | | Adapted from Rohde et al. 2019^(2)^ and Baig et al. 2018^(3)^ |
|  | **Environment** | |  | |
| Prompt | The next items will ask you about possible harms from eating red meat. |  | |  |
| Environmental harm awareness | Before today, had you ever heard that eating red meat can contribute to the following harms?  (Check the harms that you had heard about below) | [Check all that apply; randomize order of stimuli within health/environmental sections, “I haven’t heard” is always last] | | Adapted from Rohde et al. 2019^(2)^ |
| Environmental harms discouragement | 1. More greenhouse gases 2. Water shortages 3. Water pollution 4. Air pollution 5. Plants and animals going extinct 6. Clearing of forests 7. Worse land quality 8. Climate change   I haven’t heard of red meat contributing to any of these harms before [make answer exclusive] |  | |  |
| Harm discouragement – Environmental harms | Eating red meat may contribute to the harms we just asked you about.  How much does knowing that eating red meat contributes to these harms discourage you from wanting to eat red meat? | 1=Not at all  2=A little  3=Somewhat  4=A lot  5=Very much | | Adapted from Rohde et al. 2019^(2)^ and Baig et al. 2018^(3)^ |
|  | **Demographics & Health Behaviors** | |  | |
| Prompt | The next questions are about you and your household. |  | |  |
| Financial situation | How would you describe your household’s financial situation right now? Would you say that: | 1 = You are having difficulty paying the bills, no matter what you do  2 = You have money to pay the bills, but only because you have cut back on things  3 = You have enough money to pay the bills, but little spare money to buy extra or special things  4 = After paying the bills, you still have enough money for special things that you want | | DeFrank et al. 2009^(4)^ |
| Annual household income | Which of the following categories best describes your total household income in the last 12 months? It’s fine to make your best guess. | 1= Less than $10,000  2= $10,000 to $14,999  3= $15,000 to $24,999  4= $25,000 to $34,999  5= $35,000 to $49,999  6= $50,000 to $74,999  7= $75,000 to $99,999  8= $100,000 to $149,999  9= $150,000 to $199,999  10= $200,000 or more | | Population Assessment of Tobacco and Health Study 2014 |
| Number of household members who depends on this income | How many people depend on this income, including you? | # of people [restricted to 1-20] | | USDHHS 2016 |
| Education | What is the highest level of school you have completed? | 1=Less than high school or U.S. high school equivalent (GED)  2=High school diploma or U.S. high school equivalent (GED)  3=Some college  4=2-year college degree  5=4-year college degree  6=Master’s degree, graduate degree, or more | |  |
| Race of participant | What is your race? | [Select all that apply]  1=White  2=Black or African American  3=American Indian or Alaska Native  4=Asian  5=Pacific Islander  6=Some other race: ____ | |  |
| Hispanic ethnicity of participant | Are you of Hispanic, Latino, or Spanish origin? | 0 = No  1= Yes | | 2010 Census |
| Age of participant | What is your age? | _____ [validate: 18-99 allowed] | |  |
| Participant gender | What is your gender? | [Open-ended] | |  |
| Political leaning | With respect to politics, do you consider yourself to be a…. | [Randomize order of responses]  1= Liberal  2= Moderate  3= Conservative | |  |

**Supplemental Table 1.** Associations between participant characteristics and number of health harms of red meat for which participants indicated awareness, *n*=544 US parents of young children

|  | **B** | **(SE)** | ***p*** |
| --- | --- | --- | --- |
| Age in years |  |  |  |
| 18-25 | Reference | - | - |
| 26-34 | -0.28 | (0.19) | 0.145 |
| 35-44 | -0.07 | (0.20) | 0.709 |
| 45 or older | -0.02 | (0.25) | 0.923 |
| Female^a^ | -0.21 | (0.13) | 0.107 |
| Race/ethnicity |  |  |  |
| White | Reference | - | - |
| Black | **0.76** | **(0.27)** | **0.005** |
| Latino(a) | 0.11 | (0.17) | 0.509 |
| Other race/ethnicity | 0.26 | (0.18) | 0.142 |
| Education |  |  |  |
| High school or less | Reference | - | - |
| Some college | 0.43 | (0.25) | 0.084 |
| College degree | **0.56** | **(0.21)** | **0.007** |
| Graduate degree | **0.52** | **(0.25)** | **0.039** |
| Annual household income |  |  |  |
| Less than $25,000 | Reference | - | - |
| $25,000-$49,999 | 0.24 | (0.23) | 0.313 |
| $50,000-$74,999 | 0.19 | (0.22) | 0.382 |
| $75,000-$99,999 | 0.20 | (0.24) | 0.410 |
| $100,000 or more | 0.18 | (0.24) | 0.454 |
| Political leaning |  |  |  |
| Liberal | Reference | - | - |
| Moderate | **-0.33** | **(0.15)** | **0.030** |
| Conservative | -0.17 | (0.16) | 0.308 |
| Red meat consumption, servings per day | -0.09 | (0.08) | 0.256 |

*Note*. Bs are unstandardized regression coefficients from negative binomial regressions, regressing the total number of health harms for which participants reported awareness on participant characteristics. Models estimated robust standard errors. **Bold** coefficients are statistically significant, *p*<0.05.

^a^Referent group was male. The one nonbinary participant was excluded from analysis due to small cell size.

**Supplemental Table 2.** Associations between participant characteristics and number of environmental harms of red meat for which participants indicated awareness, *n*=544 US parents of young children

|  | **B** | **(SE)** | ***p*** |
| --- | --- | --- | --- |
| Age in years |  |  |  |
| 18-25 | Reference | - | - |
| 26-34 | **-0.37** | **(0.17)** | **0.028** |
| 35-44 | -0.27 | (0.18) | 0.143 |
| 45 or older | -0.01 | (0.29) | 0.979 |
| Female^a^ | **-0.33** | **(0.14)** | **0.022** |
| Race/ethnicity |  |  |  |
| White | Reference | - | - |
| Black | 0.27 | (0.25) | 0.282 |
| Latino(a) | 0.01 | (0.16) | 0.961 |
| Other race/ethnicity | -0.10 | (0.20) | 0.633 |
| Education |  |  |  |
| High school or less | Reference | - | - |
| Some college | 0.14 | (0.27) | 0.610 |
| College degree | **0.45** | **(0.21)** | **0.033** |
| Graduate degree | 0.46 | (0.25) | 0.061 |
| Annual household income |  |  |  |
| Less than $25,000 | Reference | - | - |
| $25,000-$49,999 | **0.54** | **(0.26)** | **0.038** |
| $50,000-$74,999 | **0.71** | **(0.25)** | **0.005** |
| $75,000-$99,999 | **0.63** | **(0.26)** | **0.015** |
| $100,000 or more | **0.71** | **(0.27)** | **0.008** |
| Political leaning |  |  |  |
| Liberal | Reference | - | - |
| Moderate | -0.26 | (0.15) | 0.084 |
| Conservative | **-0.54** | **(0.16)** | **0.001** |
| Red meat consumption, servings per day | **0.15** | **(0.07)** | **0.033** |

*Note*. Bs are unstandardized regression coefficients from negative binomial regressions, regressing the total number of environmental harms for which participants reported awareness on participant characteristics. Models estimated robust standard errors. **Bold** coefficients are statistically significant, *p*<0.05.

^a^Referent group was male. The one nonbinary participant was excluded from analysis due to small cell size.

**Supplemental Table 3.** Associations between participant characteristics and average discouragement from wanting to eat red meat in response to health harms of red meat, *n*=544 US parents of young children

|  | **B** | **(SE)** | ***p*** |
| --- | --- | --- | --- |
| Age |  |  |  |
| 18-25 | Reference | - | - |
| 26-34 | **-0.45** | **(0.17)** | **0.010** |
| 35-44 | **-0.38** | **(0.18)** | **0.041** |
| 45 or older | -0.15 | (0.27) | 0.577 |
| Female^a^ | 0.01 | (0.13) | 0.920 |
| Race/ethnicity |  |  |  |
| White | Reference | - | - |
| Black | **0.57** | **(0.26)** | **0.031** |
| Latino(a) | 0.23 | (0.16) | 0.134 |
| Other race/ethnicity | -0.13 | (0.21) | 0.550 |
| Education |  |  |  |
| High school or less | Reference | - | - |
| Some college | 0.06 | (0.20) | 0.742 |
| College degree | 0.26 | (0.17) | 0.139 |
| Graduate degree | 0.19 | (0.22) | 0.376 |
| Annual household income |  |  |  |
| Less than $25,000 | Reference | - | - |
| $25,000-$49,999 | 0.14 | (0.20) | 0.495 |
| $50,000-$74,999 | 0.02 | (0.21) | 0.906 |
| $75,000-$99,999 | 0.36 | (0.22) | 0.108 |
| $100,000 or more | 0.08 | (0.23) | 0.734 |
| Political leaning |  |  |  |
| Liberal | Reference | - | - |
| Moderate | -0.29 | (0.15) | 0.052 |
| Conservative | **-0.49** | **(0.15)** | **0.001** |
| Perceived health harmfulness of red meat | **0.23** | **(0.07)** | **0.002** |
| Perceived environmental harmfulness of red meat | 0.04 | (0.08) | 0.570 |
| Red meat consumption, servings per day | **0.30** | **(0.09)** | **0.001** |

*Note*. Bs are unstandardized regression coefficients from ordinary least squares regressions regressing participants’ average discouragement ratings (across all 8 health harms) on participant characteristics. **Bold** coefficients are statistically significant, *p*<0.05.

^a^Referent group was male. The one nonbinary participant was excluded from analysis due to small cell size.

**Supplemental Table 4.** Associations between participant characteristics and average discouragement from wanting to eat red meat in response to environmental harms of red meat, *n*=544 US parents of young children

|  | **B** | **(SE)** | ***p*** |
| --- | --- | --- | --- |
| Age |  |  |  |
| 18-25 | Reference | - | - |
| 26-34 | **-0.35** | **(0.17)** | **0.034** |
| 35-44 | **-0.46** | **(0.17)** | **0.009** |
| 45 or older | -0.38 | (0.25) | 0.130 |
| Female^a^ | -0.09 | (0.12) | 0.446 |
| Race/ethnicity |  |  |  |
| White | Reference | - | - |
| Black | **0.54** | **(0.25)** | **0.031** |
| Latino(a) | 0.20 | (0.15) | 0.172 |
| Other race/ethnicity | -0.27 | (0.20) | 0.177 |
| Education |  |  |  |
| High school or less | Reference | - | - |
| Some college | 0.18 | (0.19) | 0.336 |
| College degree | **0.33** | **(0.17)** | **0.048** |
| Graduate degree | **0.44** | **(0.21)** | **0.036** |
| Annual household income |  |  |  |
| Less than $25,000 | Reference | - | - |
| $25,000-$49,999 | 0.04 | (0.19) | 0.825 |
| $50,000-$74,999 | 0.11 | (0.20) | 0.578 |
| $75,000-$99,999 | 0.27 | (0.21) | 0.204 |
| $100,000 or more | 0.05 | (0.22) | 0.828 |
| Political leaning |  |  |  |
| Liberal | Reference | - | - |
| Moderate | **-0.52** | **(0.14)** | **<0.001** |
| Conservative | **-0.78** | **(0.14)** | **<0.001** |
| Perceived health harmfulness of red meat | **0.14** | **(0.07)** | **0.049** |
| Perceived environmental harmfulness of red meat | 0.09 | (0.07) | 0.217 |
| Red meat consumption, servings per day | **0.42** | **(0.08)** | **<0.001** |

*Note*. Bs are unstandardized regression coefficients from ordinary least squares regressions regressing participants’ average discouragement ratings (across all 8 environmental harms) on participant characteristics. **Bold** coefficients are statistically significant, *p*<0.05.

^a^Referent group was male. The one nonbinary participant was excluded from analysis due to small cell size.

**References**

1. Neff RA, Edwards D, Palmer A, et al. (2018) Reducing meat consumption in the USA: a nationally representative survey of attitudes and behaviours. *Public health nutrition* **21**, 1835–1844.

2. Rohde JA, Noar SM, Mendel JR, et al. (2019) E-Cigarette Health Harm Awareness and Discouragement: Implications for Health Communication. *Nicotine & Tobacco Research*.

3. Baig SA, Noar SM, Gottfredson NC, et al. (2018) UNC Perceived Message Effectiveness Scale (UPMES): Development and validation of a brief scale. vol. 52, pp. S78–S78. Oxford University Press.

4. DeFrank JT, Rimer BK, Gierisch JM, et al. (2009) Impact of mailed and automated telephone reminders on receipt of repeat mammograms: A randomized controlled trial. *Am J Prev Med* **36**, 459–467.
